# Leading cancers contributing to educational disparities in cancer mortality in the US, 2017

**DOI:** 10.1007/s10552-021-01471-9

**Published:** 2021-07-09

**Authors:** Diana R. Withrow, Neal D. Freedman, James T. Gibson, Mandi Yu, Anna M. Nápoles, Amy Berrington de González, Meredith S. Shiels

**Affiliations:** 1 Division of Cancer Epidemiology & Genetics, National Cancer Institute, National Institutes of Health, Rockville, MD USA; 2 Information Management Services Inc, MD, USA; 3 Surveillance Research Program, Division of Cancer Control & Population Sciences, National Cancer Institute, National Institutes of Health, Rockville, MD USA; 4 Division of Intramural Research, National Institute for Minority Health and Disparities, National Institutes of Health, Bethesda, MD, USA

**Keywords:** Cancer mortality, Disparities, Lung cancer, Risk difference, Education

## Abstract

**Purpose:**

To inform prevention efforts, we sought to determine which cancer types contribute the most to cancer mortality disparities by individual-level education using national death certificate data for 2017.

**Methods:**

Information on all US deaths occurring in 2017 among 25–84-year-olds was ascertained from national death certificate data, which include cause of death and educational attainment. Education was classified as high school or less (≤ 12 years), some college or diploma (13–15 years), and Bachelor's degree or higher (≥ 16 years). Cancer mortality rate differences (RD) were calculated by subtracting age-adjusted mortality rates (AMR) among those with ≥ 16 years of education from AMR among those with ≤ 12 years.

**Results:**

The cancer mortality rate difference between those with a Bachelor's degree or more vs. high school or less education was 72 deaths per 100,000 person-years. Lung cancer deaths account for over half (53%) of the RD for cancer mortality by education in the US.

**Conclusion:**

Efforts to reduce smoking, particularly among persons with less education, would contribute substantially to reducing educational disparities in lung cancer and overall cancer mortality.

**Supplementary Information:**

The online version contains supplementary material available at 10.1007/s10552-021-01471-9.

Within the United States, disparities in cancer mortality rates by socio-economic status are large and have increased over recent years [1]. To inform prevention efforts, we examined differences in mortality rates from the 15 leading causes of cancer death by educational level and examined how disparities for each cancer contributed to the disparity observed overall.

Information on all US deaths occurring in 2017 among 25–84-year-olds was ascertained from national death certificate data, which include cause of death and educational attainment [2]. Education was classified as high school or less (≤ 12 years), some college or diploma (13–15 years), and Bachelor's degree or higher (≥ 16 years). Cancer deaths were classified based on ICD-10 codes.

Population denominators were linearly extrapolated from the American Community Survey (ACS) 1% Integrated Public Use Microdata for 2006–2014, stratified by education, age, sex and race/ethnicity. Extrapolation was used because this was the most recent ACS data available at the time of preparation of the dataset. Cancer mortality rate differences (RD) were calculated by subtracting age-adjusted mortality rates (AMR) among those with ≥ 16 years of education from AMR among those with ≤ 12 years.

Cancer mortality rates in 2017 were 47% higher among persons with ≤ 12 years of education (AMR: 224.7/100,000 person-years, Supplementary Fig. 1) than among people with ≥ 16 years (AMR: 152.6; RD:72.3/100,000, Fig. 1), with larger differences observed among men (RD: 117.5) than women (RD: 45.2).

By site, lung cancer was the largest contributor to the overall RD in both men and women, accounting for half of the difference (RD_male_: 54.9, RD_female_: 27.5, Fig. 1). Among men, the next leading contributors were liver (RD: 10.5), colorectal (RD: 10.2) and esophageal cancers (RD: 5.1) Among women, the next leading contributors were colorectal (RD: 4.0), liver (RD: 2.2) and stomach cancers (RD: 1.2). Although breast and prostate cancers are the second and third leading causes of cancer mortality in women and men respectively, the RDs by educational level for these cancers were small (RD_breast_: -0.7, RD_prostate_: 3.9).

**Fig. 1:**
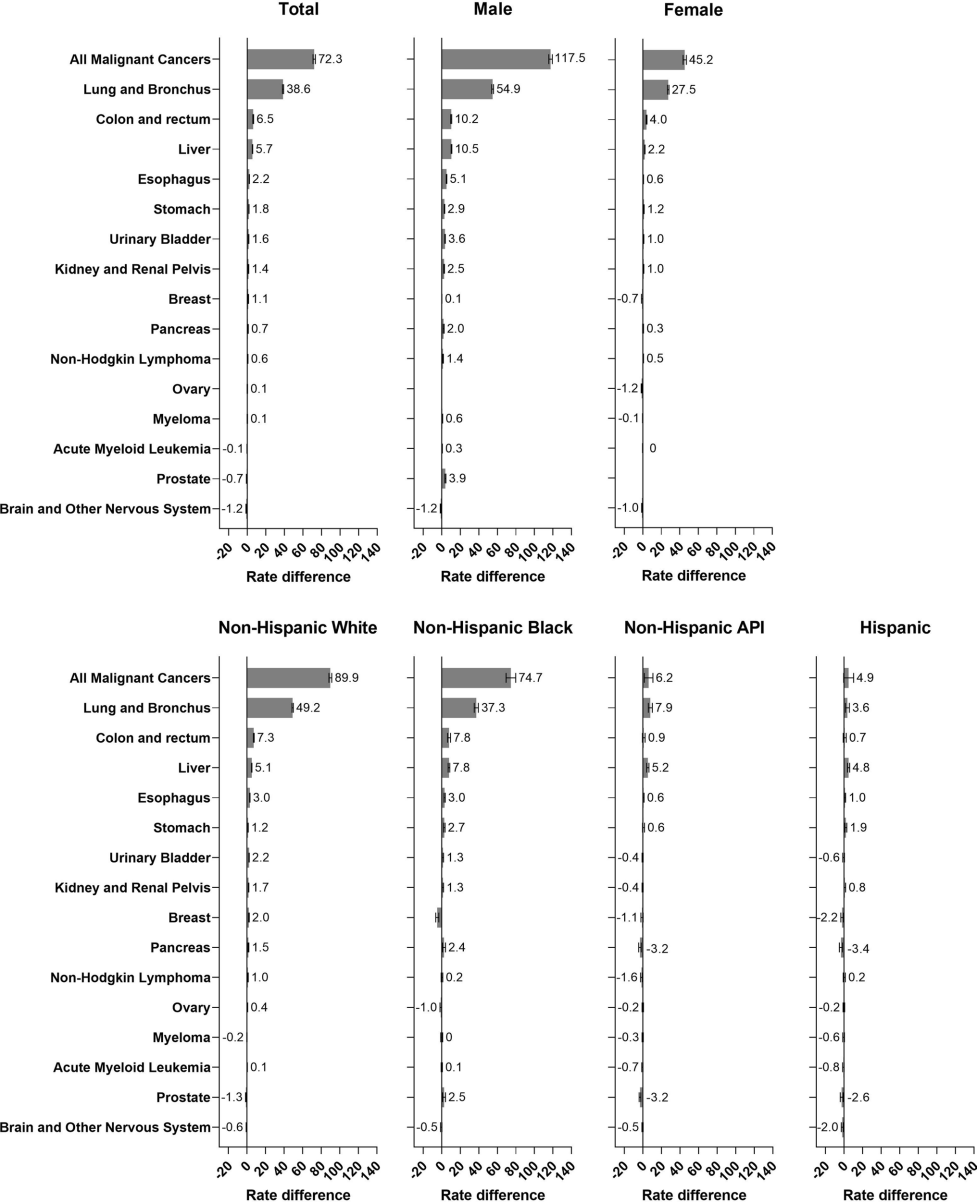
Cancer mortality rate differences, ≥ 16 years vs. ≤ 12 years of education, stratified by sex and race/ethnicity; all 2017 deaths among persons aged 25–84 in the US; rate differences are age-standardized to the 2000 US standard population and are expressed per 100,000 person-years and 95% confidence intervals are displayed; API: Asian and Pacific Islander

Cancer mortality RDs by educational attainment were higher for non-Hispanic white (RD: 89.9) and non-Hispanic black adults (RD: 74.7) than among Asian/Pacific Islander (RD: 6.2) and Hispanic adults (RD: 4.9). Exploratory analyses suggested that the RDs among Asian/Pacific Islander and Hispanic adults were driven by differences in mortality rates by education among men, more so than among non-Hispanic white and black adults, where patterns were similar by sex (not shown).

Lung cancer deaths account for over half (53%) of the RD for cancer mortality by education in the US. Cigarette smoking causes most lung cancers and profound educational disparities in smoking have been noted in the US [3]. National Health Interview Survey 2017 data indicate that 5.9% of people with a Bachelor’s degree or more smoke vs. 24.9% of people with less than a high school education [4]. Lung cancer was the largest contributor to disparities in all racial/ethnic groups, except for Hispanic adults, likely due to smaller education-related disparities in smoking behavior within Hispanic populations than white populations [5, 6]. Smoking is a risk factor for 7 of 15 leading causes of cancer death, thus, eliminating disparities in cigarette smoking would do much to eliminate educational disparities in US cancer mortality rates.

Strengths of our analysis include use of data on all deaths occurring in the country and stratification of mortality rates by sex, race/ethnicity, and education. Because of the large population size, any error imposed by sampling variation in estimating the populations using the ACS was negligible [7]. The primary limitation of this work is that education may be misreported on death certificates, and this may be differential by race/ethnicity and age. In the past, Black and Hispanic high school graduates and older decedents were most likely to have their education under-reported [8, 9]. Additionally, while all other states used the 2003 revised US Standard Death Certificate which assessed highest degree completed, West Virginia transitioned from the 1999 version, assessing years of education, to the 2003 version over the course of 2017 [10]. By aggregating persons with high school and less education, we minimized the impact of these potential misclassifications.

Our results underscore that educational disparities in smoking contribute substantially to disparities in lung cancer and overall cancer mortality. Reductions in smoking prevalence would also reduce deaths from many smoking-associated diseases and cancer types, saving millions of lives annually [11, 12].

## Supplementary Information

Below is the link to the electronic supplementary material.

Supplementary file1 (DOCX 85 KB)

## Data Availability

Not applicable.

## Abbreviations

*AMR*: Age-adjusted mortality rate
*RD*: Rate difference
