# Supplementary material for: Leading cancers contributing to educational disparities in cancer mortality in the US, 2017
Source: Cancer Causes Control. 2021 Jul 9;32(11):1193–6. doi: 10.1007/s10552-021-01471-9 (PMC8492562; doi:10.1007/s10552-021-01471-9)
Supplement: Supplementary file 1 — Supplementary file1 (DOCX 85 KB) [file 10552_2021_1471_MOESM1_ESM.docx]

**SUPPLEMENTARY RESULTS**

**
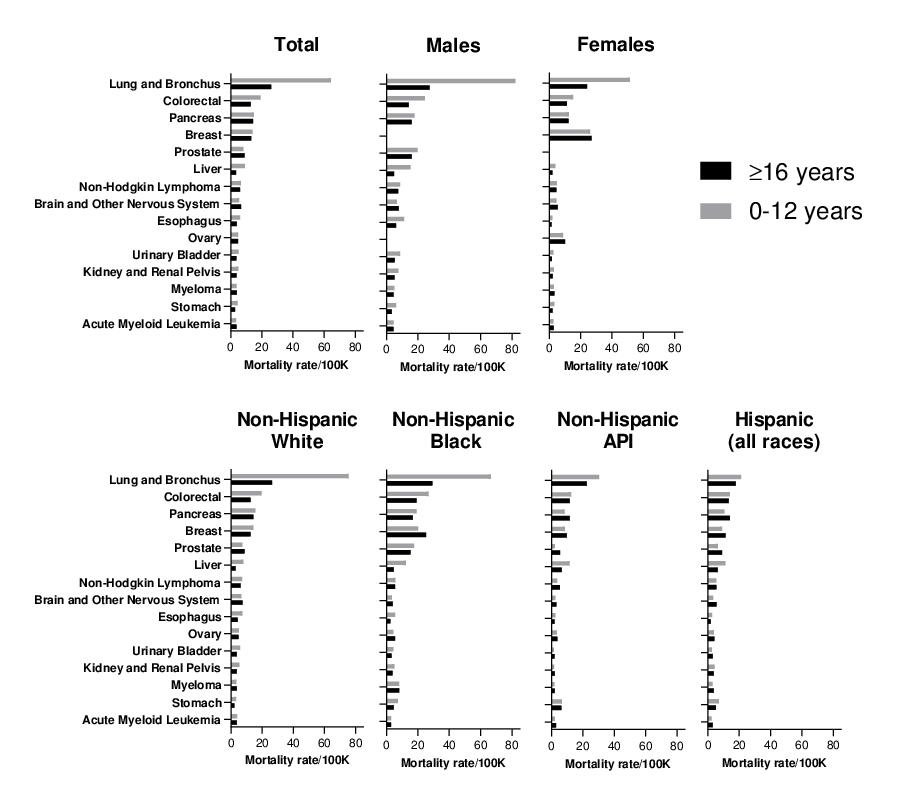
**

**Supplementary Figure 1.** Cancer mortality rates stratified by sex, race/ethnicity and years of education (≥16 years vs. ≤12 years); all 2017 deaths among persons aged 25-84 in the US; rates are age-standardized to the 2000 US standard population and are expressed per 100,000 person-years; API: Asian and Pacific Islander
